# Obstructive Sleep Apnea Syndrome (OSAS). Review of the literature

**DOI:** 10.4317/medoral.17706

**Published:** 2012-05-01

**Authors:** Eva Azagra-Calero, Eduardo Espinar-Escalona, José M. Barrera-Mora, José M. Llamas-Carreras, Enrique Solano-Reina

**Affiliations:** 1 Master of Orthodontics and Dentofacial Orthopedics. University of Seville; 2 Professor of the Master of Orthodontics and Dentofacial Orthopedics.School of Dentistry.University of Seville; 3 Chairman of Orthodontics. Stomatology Department. University of Seville. Seville, Spain

## Abstract

Obstructive sleep apnea and hypopnea syndrome is characterized by repeated airway collapse during sleep. The li-terature describes multiple causes of the disease. The main cause is a reduction of the expansion forces of the pharyngeal dilator muscles, as in situations of genioglossal muscle dysfunction, and discoordination between the inspiratory activity of the muscle and respiratory effort, which play an important role in progression of the disease. Other described causes are soft tissue disorders, such as macroglossia or tonsillar hypertrophy, and skeletal structural alterations such as micrognathia and retrognathia. The syndrome is also more frequent in obese people, where the accumulation of fat in the neck region produces narrowing of the pharyngeal airway, thereby diminishing the passage of air. This review focuses on the pathogenesis, epidemiology, main features and diagnosis of the disease, and on its main forms of treatment.

** Key words:**Sleep apnea, obstructive sleep apnea, sleep apnea syndrome, obstructive sleep apnea syndrome.

## Concept

Obstructive sleep apnea syndrome (OSAS) is characte-rized by episodes of partial or complete obstruction of the upper airway during sleep, interrupting (apnea) or reducing (hypopnea) the flow of air, followed by transient awakening that leads to the restoration of upper airway permeability.

These cycles of apnea/hypopnea are repeated several times everyhour, producing fragmented and scantly repairing sleep.

Within the upper airway, the pharynx, and particularly the oropharynx and hypopharynx, is the region where most obstructive processes leading to OSAS are found (1). OSAS has a negative impact on the health and behavior of millions of adolescents throughout the world. It is an independent risk factor for many diseases, such as hypertension, heart failure, heart attack, cardiovascular eventsand arrhythmias. Unfortunately, it isa common chronic disease that greatly conditions the life of the patient (2).

Breathing disorders related with sleep are studied and treated in sleep pathology units involving different specialists. Orthodontists can make an important contribution to the diagnosis and management of these diseases, and have recently also been incorporated to such units.

The contribution of orthodontists to the study and treatment of respiratory disorders associated with sleep focuses onthree aspects.

a) Diagnosis of the structural changes often present in these diseases.

b) The treatment of mild to moderate forms usingintraoral appliances.

c) Presurgical orthodontic treatment of patients programmed for orthognathic surgery (2).

## Epidemiology

OSAS is the second disease in order of frequency among the different respiratory disorders, surpassed only by asthma.

The syndrome can affect any age group, and is estimated to affect 2-4% of the adult population, though it is more common in middle aged males. One out of every 5 adults suffers moderate OSAS, and one out of every 15 presents moderate to severe OSAS (3).

The syndrome is characterized by tense breathing, decreased levels of oxygen in blood, and arousals that disrupt normal sleep (1).

Some cases posea high risk to health, and patients may experience excessive sleepiness during the day, headaches in theearly morning, impaired concentration, social problems, and systemic disorders(1,2).

## Pathogenesis

Several factors are implicated in the development of OSAS (4). The main cause described in the literature is a reduction of the expansion forces of the pharyngeal dilator muscles, as in situations of genioglossal muscle dysfunction, and discoordination between the inspiratory activity of the muscle and respiratory effort (5), which play an important role in progression of the disease.

Additional factors are excessive or elongated tissues of the soft palate, macroglossia, tonsillar hypertrophy, and a redundant pharyngeal mucosa (6).

Pae et al. found tongue shape in patients with OSAS to bedifferent from that ofnormal subjects in the supine position this being the first study to evaluate tongue shape in the supine position. Tongue shape therefore may be taken to play an important role in the development of OSAS (7).

Altered upper airway anatomy, conditioned by skeletal abnormalities as in Pierre Robin syndrome (8), or by alterations of the soft tissues of the neck, particularly in obese patients, with increased adipose tissue in the region of the neck with fat infiltration and edema in the soft palate (9), are also implicated in this syndrome.

Although obesity is regarded as a principal risk factor in the occurrence of OSAS, it has been shown that the neck perimeter is more closely correlated to severity of the syndrome than body mass index, though there is usually direct proportionality between obesity and neck perimeter (9).

## Main features of OSAS

With regard to the major signs and symptoms of OSAS, a distinction can be made between day and night.

-Nocturnal symptoms

The most frequent and characteristic nocturnal symptoms of OSAS are snoring and observed apneas, both of which reflect the underlying physiopathological events: critical narrowing of the upper airway during sleep and intermittent airway collapse, usually reported to the doctor by the couple of the patient.

Snoring

Snoringis the most common symptom of OSAS (present in up to 95% of all patients) (10). However, it is also very common in the adult general population, affecting 25-30% of all women and 40-45% of all men on a regular basis (11).

This explains why snoring is of little diagnostic value in identifying OSAS (10).

Patients who consult with suspected OSAS tend to have a long prior history of snoring that has become increasingly intense and irregular over time, often in connection with increased body weight, alcohol consumption or muscle relaxant drugs, or with menopause in women (12).

Observed apneas

Apneas are a frequent cause of consultation, since they often worry the couple of the patient, describing them as respiratory pauses that interrupt snoring while the patient continues to make efforts to breathe. Observed apneas are more predictive of a high apnea/hypopnea index (AHI) than either snoring or excessive daytime sleepiness.

Arousals

Arousals or awakenings are less frequent than observed apneas. Such situations correspond to “perceived apneas” that endwhen arousal occurs. They are conscious phenomena and are accompanied by brief and intense dyspnea sensation.

This symptom is related tohigh blood pressure (3), because repeated arousals are linked to sympathetic discharges that raiseblood pressure and heart rate.

Choking, diaphoresis, nocturia, restless sleep and somniloquy are additional nocturnal symptoms related to OSAS.

-Daytime symptoms

Daytime sleepiness

Sleepiness is the most important daytime symptom of OSAS, and is due to the fragmentation of sleep caused by recurrent electroencephalographic awakening that usually terminate the apneas and hypopneas (10).

Daytime sleepiness is of scant diagnostic value, because a number of situations and disease processes can cause the same symp-toms (12).

Morning headaches, apathy, depression, concentration difficulties, memory loss and decreased libido are other characteristic daytime symptoms of patients with OSAS, all as a consequence of daytime sleepiness.

## Diagnosis

An essential requirement for a correct diagnosis of OSAS is a correct anamnesis, recording the family history (history of OSAS) and personal antecedents (tonsillectomy/adenoidectomy in childhood, alcohol intake, the use of muscle relaxant drugs, obesity, etc). It is also important to establish the profession of the patient, since in some professions OSAS constitutes a medical emergency (12).

A proper physical examination is also required (height, weight, body mass index, cardiovascular evaluation), including exploration of the upper airway (19) (nasal passages, oropharynx and hypopharynx, and larynx).

The above data in turn should be complemented by radiological study in the form of either conventional lateral X-rays (13) or a tridimensional X-ray study (14), which will reveal the craniofacial anatomical alterations predisposing to OSAS (15).

Many tests are available for evaluating sleep and for diagnosing OSAS. The most widely used technique is polysomnography (PSG) (16), which monitors the sleeping state, respiration, electrocardiogram, movements of the legs, oximetry and snoring. In addition, PSG records the distribution of the stages of sleep, the number of awakenings, the number of apneas or hypopneas, the starting time of sleep, and the hours of efficient sleep(hours asleep/hours in bed). PSG also provides the apnea / hypopnea index (AHI); in this context, apnea is very serious and can only be treated surgically when AHI >30, while AHI 15-30 defines moderate apnea, and an AHI score of < 15 indicates mild apnea.

While PGS provides a lot of information, it is a complex and expensive technique – this limiting its practical applicability to the evaluation and treatment of OSAS.

For this reason, simple tests have been developed and are now used in many healthcare systems. While such techniques provide less information, they are cheaper and can be applied in the home of the patient (17).

## Main treatment options

Regarding the treatment of OSAS, emphasis firstly must be placed on the importance of behavioral modifications on the part of patients with OSAS, including the adoption of a regular sleep schedule, ensuring a good environment for adequate sleep, not lying down without the need to sleep, and the avoidance of too much time in bed. Secondly, alcohol consumption and smoking should be avoided. In this context, smoking increases inflammation of the upper airway and implies a greater risk of snoring and OSAS (18). Alcohol consumption in turn is associated with exacerbation of the number and duration of apneas, arterial desaturation and sleep fragmentation (19).

The American Association of Sleep Disorders (20) has proposed the use of oral appliances in order to eliminate snoring or sleep apnea, classifying them as follows: mandible advancement appliances, lingual retainers, appliances that act upon the soft palate, and combined advancement and positive pressure appliances.

Mandible advancement appliances are mostly manufactured with an advance of 80% of maximum protrusion (21). There are monoblock types and devices manufactured with two splints – no differences in success rate being observed between the two designs (22). According to the literature, the success of these appliances is associated with lower AHI scores as established by PSG (23).

Regarding the side effects of these appliances, various authors have reported pain in the upper and lower incisors, joint discomfort, dental or facial muscle discomfort, excessive salivation, dryness of the mouth, headache and bruxism, while other authors have reported only minimum dental and orthopedic effects following the use of these appliances (24,25).

Comparison of these appliances with continuous positive airway pressure (CPAP) shows them to be less effective in reducing the AHI score (26).

CPAP was introduced by Sullivan in 1981 (27). In this form of treatment, the patient wears a mask at night, attached to a machine that continuously impels air into the airway. This increases the air pressure in the pharynx; air forces the soft palate to move forwards against the tongue, and the upper airway thus receives pressure and does not collapse.

This technique requires great effort on the part of the patient, but is also the most effective form of treatment, as it has been shown to reduce the AHI score to less than 5 events per hour in most patients - with improvements in both objective and subjective sleep, and in the cardiovascular results (28). However, while very effective, many patients cannot tolerate CPAP every night for life; its acceptance is therefore rather low (28).

When non-surgical techniques for the treatment of OSAS fail or are unacceptable to patients, surgery is considered. The main options are briefly described in the ASDA 1996, and are the following (29).

-Tracheotomy

Tracheotomy was the first surgical technique described for the treatment of OSAS. In patients subjected to this technique, the tracheal hole is not kept open throughout the day, and a plug is used to allow the patient to breathe through the nose and mouth. The plug in turn is removed at night so that the air penetrates directly into the distal portion of the trachea (28).

Uvulopalato-pharyngoplasty (UPPP)

UPPP, initially described by Fujita et al., is used to correct obstruction atoropharyngeal level by modifying the uvula, removing the increased palatal or pharyngeal tissue, and primary closing the posterior and anterior pillars to expand the retropalatine tract (30).

Kamami was the first to describe laser-assisted uvulopalatoplasty(LA-UPP) in the 1980s to reduce the uvula and the distal portion of the soft palate without total excision of the muscle of the uvula (31).

This technique constituted first choice treatment. However, it is only able to improve the condition in 50% of all OSAS patients. Friedman et al. reported a 41% success rate with this technique (32).

-Tonsillectomy

Although rare in adults, hypertrophic tonsils can be an important factor in OSAS. In severe cases of obstructive tonsils, tonsillectomy at any age provides benefits and has a positive impact on the obstruction of the upper airway (32). Krespi and Ling have described several tonsillectomy procedures with the CO2 laser. The tonsillar tissue is removed in less time, with less bleeding, and with less postoperative pain compared with traditional tonsillectomy, though this procedure has been associated with a higher tonsillar tissue recurrence rate (33).

Radiofrequency ablation of the tonsils is another technique based on energy transfer through electromagnetic radiation to generate heat within the tissues. It is used to achieve a reduction in tissue volume followed by healing, and can be accompanied by similar treatment of the base of the tongue. Clinical trials suggest less pain with this procedure than with total resective surgery (34).

-Osteotomy of maxillo-mandibular advancement (mma)

This is currently the first choice treatment for OSAS. By advancing the mandible, forward repositioning is carried out of muscles such as the anterior digastric muscle, the mylohyoid muscle, genioglossal muscle and geniohyoid muscle – stretching the tongue forwards and away from the pharynx.

Moving the jaw stretches the palatine tissue, which in turn exerts traction upon the palatoglossal muscle and increases lingual support, favoring pharyngeal patency (35).

The success rate with MMA is 75-100%; the AHI score is improved over the long term even in patients who gain some weight, and the results remain stable over time (36).

In 2008 Lye et al. reported a high success rate in relation to breathing as well as in the quality of life of patients subjected to bimaxillary advancement (37).With regard to the indications for MMA surgery in these patients, the AHI score must be > 15, with low oxygen saturation (<90%) and excessive daytime sleepiness. In addition, conservative treatments such as weight loss, mandibular advancement and/or CPAP devices should be found to be poorly tolerated or ineffective in these patients in order to choose the surgical option of MMA. On the other hand, two characteristics must be fulfilled in order to indicate MMA:

(a) Firstly, there must be multiple sites of obstruction, or blockage must be diffuse and inaccessible.

(b) Secondly, the patient must present skeletal class II malocclusion, and MMA surgery must offer multiple benefits for the patient(38).

An additional procedure for completing MMA is ge-nioglossal muscle advancement (GA). This can be done with a horizontal osteotomy technique popularized by Riley et al., or a lower horizontal geniotomy; the standard chin osteotomy used in orthog-nathic surgery. This technique expands the magnitude of genioglossal, ge-niohyoid and digastric muscle replacement(39).

Conley et al. published the case of a patient with OSAS subjected to transverse osteogenic distraction of the ma-xilla combined with maxillo-mandibular advancement – followed by significant improvement of the apnea (40).

MMA is therefore an efficient technique for the treatment of OSAS patients, given the results of various studies that have exam-ined the airways of patient with OSAS subjected to this surgical procedure - revealing greater posterior and lateral airway dimensions, with minimal complications after the operation(37).

## Conclusions

- OSAS is a disease that affects millions of people, and is linked to cardiovascular consequences and stroke.

- OSAS can be successfully treated in most cases, following the recommended treatment guidelines.
